# Unraveling dual fusion mechanisms in BmNPV GP64: critical roles of CARC motifs and signal peptide retention

**DOI:** 10.1128/jvi.01511-24

**Published:** 2024-11-27

**Authors:** Luping Sun, Ying Xu, Kai Chen, Wenbin Nan, Meixian Wang, Yiling Zhang, Bifang Hao, Jinshan Huang

**Affiliations:** 1 Jiangsu Key Laboratory of Sericultural and Animal Biotechnology, School of Biotechnology, Jiangsu University of Science and Technology 12676 , Zhenjiang, China; 2 Key Laboratory of Silkworm and Mulberry Genetic Improvement, Ministry of Agriculture and Rural Affairs, Sericultural Scientific Research Center, Chinese Academy of Agricultural Sciences 625370 , Zhenjiang, China; The University of Arizona, Tucson, Arizona, USA

**Keywords:** BmNPV, GP64, baculovirus, CARC, membrane fusion, signal peptide

## Abstract

Viral membrane fusion is a critical process enabling viruses to invade host cells, driven by viral membrane fusion proteins (MFPs). Cholesterol plays a pivotal role in this process, which is essential for the infectivity of many enveloped viruses. The interaction between MFPs and cholesterol is often facilitated by specific amino acid motifs known as cholesterol recognition/interaction amino acid consensus (CRAC) motifs and reverse CARC motifs. In a previous study, we demonstrated that CRAC1 and CRAC2 in GP64 are required for Bombyx mori nucleopolyhedrovirus (BmNPV) infection. This study further investigates the role of CARC in the GP64 protein of BmNPV, revealing their complex interaction with cholesterol and the influence of signal peptide (SP) retention on viral infectivity. We identified six putative CARC motifs in GP64 and generated mutants to assess their function. Our findings show that CARC1, CARC2, CARC3, and CARC4 are indispensable for viral fusion and infection when the SP is retained, whereas only CARC2 and CARC3 remain essential after SP cleavage. In contrast, CARC1 and CARC4 are necessary for viral infection through a cholesterol-independent mechanism resulting from double mutations in the CRAC1 and CRAC2 motifs of GP64. These insights not only deepen our understanding of BmNPV GP64-mediated fusion but also highlight potential antiviral targets, underscoring the adaptability and resilience of viral fusion mechanisms.

**IMPORTANCE:**

Understanding viral membrane fusion mechanisms is crucial for developing antiviral strategies. This study provides novel insights into the intricate roles of CARC and CRAC motifs in the GP64 protein of BmNPV, particularly their interaction with cholesterol and the influence of signal peptide retention. The discovery that certain CARC motifs are essential for cholesterol-dependent fusion, whereas others function in a cholesterol-independent context advances our understanding of viral fusion processes. These findings emphasize the potential of targeting CARC motifs for therapeutic interventions and underline the importance of cholesterol interactions in viral infections. This research not only deepens our understanding of BmNPV fusion mechanisms but also has broader implications for other enveloped viruses.

## INTRODUCTION

The baculovirus envelope protein GP64 is a well-characterized membrane fusion protein (MFP) that plays a crucial role in the virus’s ability to infect host cells by mediating membrane fusion through fusion loops (FLs) (1, 2). Despite sharing 90% genomic identity, Autographa californica multicapsid nucleopolyhedrovirus (AcMNPV) and Bombyx mori nucleopolyhedrovirus (BmNPV) have distinct host ranges (3, 4). AcMNPV infects a broader spectrum of insects and cell lines, including *Bombyx mori* and its cells (5–7), whereas BmNPV replicates in *Bombyx mori* but not in *Spodoptera frugiperda* cell lines (Sf21, Sf9), which are permissive for AcMNPV (3). This narrow host range of BmNPV is attributed to the insufficient functionality of its GP64 protein (6, 8). Notably, BmNPV GP64 retains its signal peptide (SP), which determines its secretion, cholesterol-dependent fusion, and viral infectivity (9, 10). Understanding the mechanisms by which GP64 facilitates membrane fusion is essential not only for elucidating BmNPV infectivity but also for broader applications in virology, including the design of viral entry inhibitors and gene delivery vectors.

Previous studies have established that GP64-mediated fusion is highly dependent on the presence of cholesterol in the host cell membrane (9, 11–13), a characteristic shared by many viral fusogens (14). Cholesterol-rich microdomains, or lipid rafts, in the host membrane are believed to be critical sites for viral entry (15–22), providing the necessary environment for fusogen activation and membrane fusion. However, the precise molecular interactions between GP64 and host membrane components, particularly cholesterol, remain incompletely understood.

The interaction between MFPs and cholesterol is often mediated by specific amino acid motifs known as cholesterol recognition/interaction amino acid consensus (CRAC), defined by the consensus sequence (L/V)-X_1-5_-Y-X_1-5_-(K/R), or its reverse (CARC) with the algorithm (R/K)-X_1-5_-(F/Y/W)-X_1-5_-(L/V) (23). CARC motifs preferentially interact with the outer membrane leaflet, whereas CRAC motifs favor the inner leaflet (24). These motifs are hypothesized to interact directly with cholesterol during viral infection (23, 25–27), thereby facilitating the fusion process. In BmNPV GP64, our previous study identified CRAC1 and CRAC2 domains are critical for infection (9). However, only CRAC2 domain is essential when the SP is cleaved from BmNPV GP64 (n-region deletion results in SP cleavage from GP64), which is the same CRAC domain present in AcMNPV GP64 (9, 12). The retention of the SP in GP64 has been suggested to influence its fusion capabilities (9, 10, 28, 29), although the specific contributions of SP retention to the fusion mechanism remain to be fully elucidated.

The double CRAC mutation in BmNPV GP64 unexpectedly restores infectivity in a cholesterol-independent manner, likely due to the presence of uncleaved SP (9). Although the CRAC motif is the first consensus sequence known to bind cholesterol, thermodynamic analyses indicate that the CARC motif generally exhibits a stronger affinity for cholesterol (30). Although previous research has established the cholesterol dependence of GP64-mediated fusion, the specific roles of CARC motifs in this process remain unclear. Our study aims to dissect the role of these CARC and CRAC motifs in the context of BmNPV GP64-mediated fusion, with a particular focus on how SP retention affects their function during viral entry. We hypothesize that BmNPV GP64 utilizes a dual-pathway fusion mechanism—cholesterol-dependent and independent—modulated by specific CARC and/or CRAC motifs and SP retention, to adapt to variations in host cell membrane composition.

To test this hypothesis, we performed a series of mutational analyses targeting the CARC and CRAC motifs within GP64 and examined the effects of these mutations on viral infectivity and membrane fusion. Additionally, we employed structural predictions to visualize the spatial arrangement of these motifs within the GP64 trimer and better understand their functional roles. Our findings reveal a complex interplay between cholesterol-dependent and independent fusion mechanisms, mediated by distinct CARC motifs in GP64. These insights not only deepen our understanding of BmNPV infectivity but also suggest potential strategies for disrupting viral entry, which could be applicable to other viruses that rely on similar fusion mechanisms.

## RESULTS

### CARC1, CARC2, CARC3, and CARC4 are required for BmNPV infectivity with SP-uncleaved GP64

**Fig 1:**
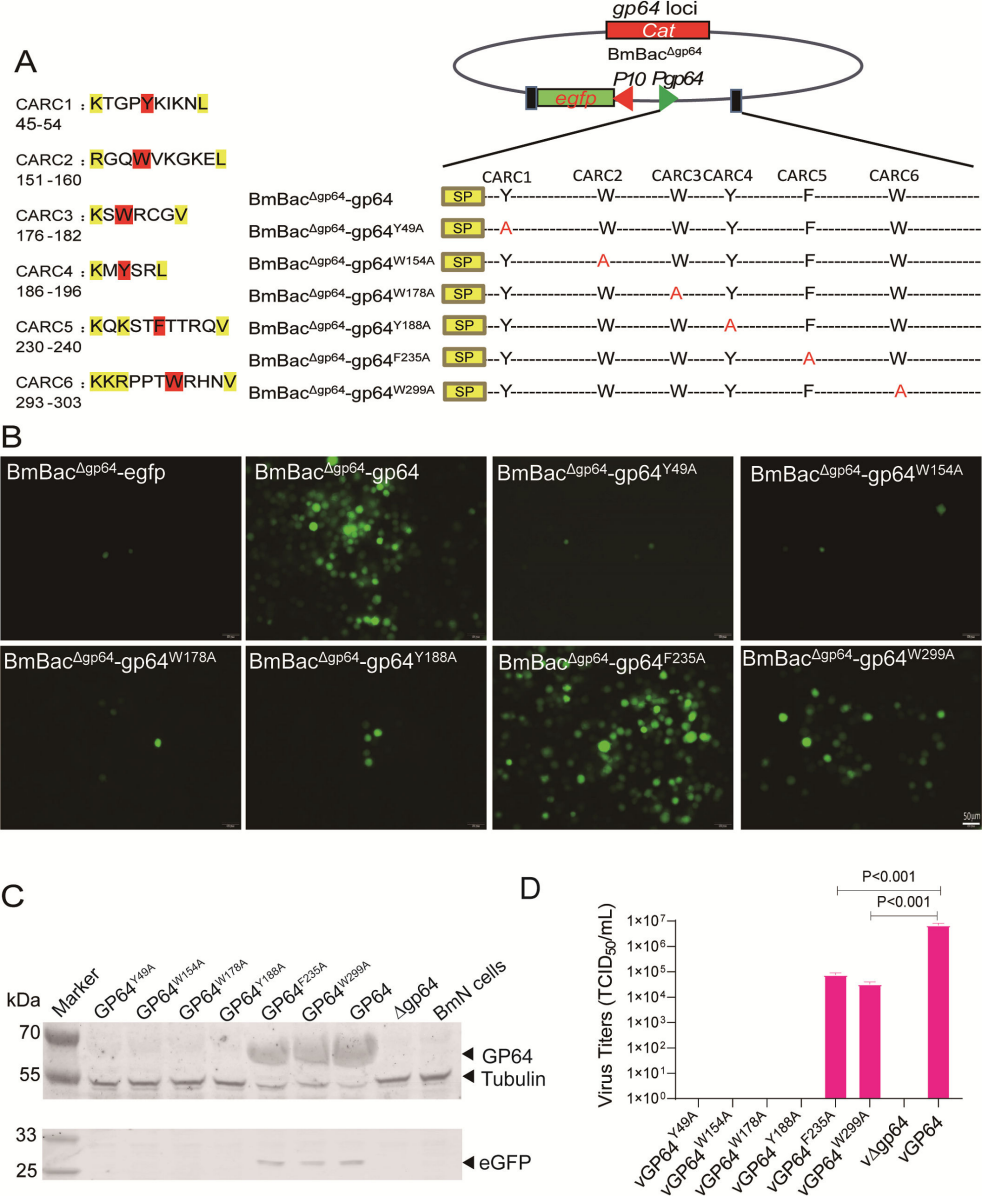
Mutation analysis of CARC in wild-type GP64. (**A**) Prediction of CARC sites in the ectodomain of BmNPV GP64 and schematic representation of bacmids containing CARC mutations. Aromatic amino acids are highlighted with a red background and replaced by alanine (red letters). These mutants and *egfp* drived by the P10 promoter were inserted into the *gp64*-null bacmid at the *ph* locus. (**B**) Virus replication analysis in BmN cells. Recombinant bacmids were transfected into BmN cells using H4000 transfection reagent, following the protocol. Fluorescence microscopy images were captured at 96 h p.t. Scale bar: 50 µm. (**C**) Western blot analysis of CARC-mutated GP64 in BmN cells. Transfected cells were subjected to SDS-PAGE and western blotting using GP64, β-Tubulin, and eGFP antibodies. (**D**) Viral titer comparison of CARC mutants. Supernatants from transfected cells were collected at 120 h p.t. for titration by EPDA.

We identified six putative CARC motifs in BmNPV GP64 ectodomain based on the consensus sequence: CARC1 (residues 45–54), CARC2 (residues 151–160), CARC3 (residues 176–182), CARC4 (residues 186–196), CARC5 (residues 230–240), and CARC6 (residues 293–303) (Fig. 1A). To assess their roles in BmNPV infection, we generated six *gp64* mutants driven by *gp64* promoter, incorporating alanine substitutions at key aromatic residues through overlapping PCR. An *egfp* marker was inserted downstream of the *P10* promoter, a very late gene of BmNPV. These genes were then transposed into the *polyhedron* loci of a *gp64*-null bacmid (BmBac^Δgp64^), producing recombinant bacmids (Fig. 1A). Wild-type *gp64* and *egfp*-repaired bacmid (BmBac^Δgp64^-gp64) and an *egfp*-only repaired bacmid (BmBac^Δgp64^-egfp) served as controls. Mutations in CARC1 (Y49A), CARC2 (W154A), CARC3 (W178A), and CARC4 (Y188A) led to a significant loss of viral infectivity, demonstrating their essential role in BmNPV infection. In contrast, mutations in CARC5 (F235A) and CARC6 (W299A) reduced, but did not completely eliminate, infectivity, suggesting they are not essential for viral infection (Fig. 1B). Western blot analyses showed substantial amounts of GP64 and eGFP in cells expressing GP64^F235A^, GP64^W299A^, and GP64, indicating virus amplification (Fig. 1C). Viral titers confirmed that CARC1-4 mutations completely abolished infectivity, whereas CARC5 and CARC6 mutations significantly reduced, but did not eliminate, viral infectivity (Fig. 1D).

### CARC mutations impact GP64 fusogenicity

**Fig 2:**
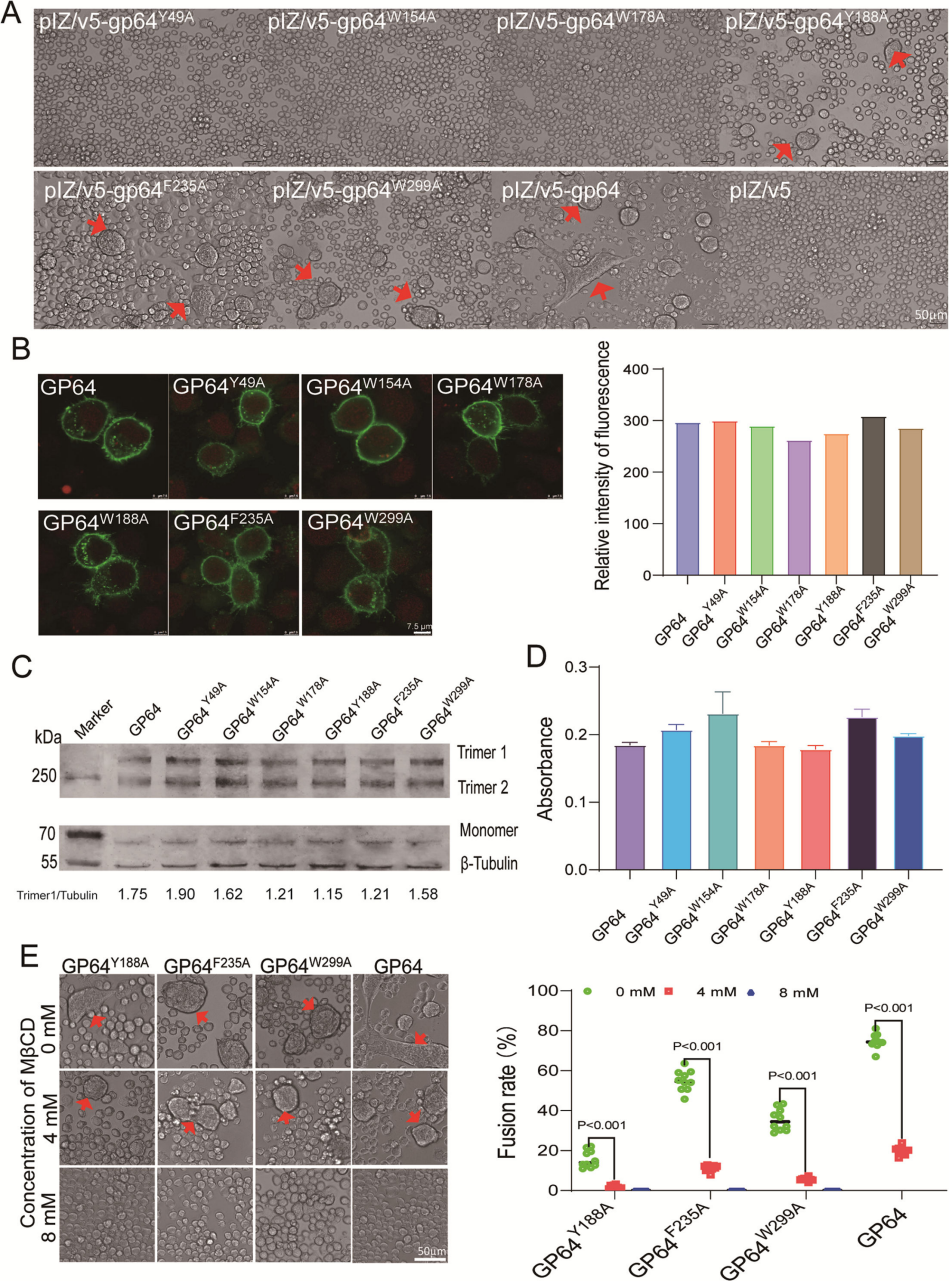
Expression analysis of CARC mutants. (**A**) Cell-cell fusion assay. BmN cells in a 24-well plate were transfected with 0.8 µg of plasmid DNA and incubated at low pH for 5 min to induce fusion at 72 h p.t. Arrows indicate syncytia. Scale bar: 50 µm. (**B**) Immunofluorescence analysis. BmN cells were transfected with 2 µg of plasmid containing the CARC mutants and fixed for immunofluorescence staining at 72 h p.t. using anti-GP64 antibody and FITC-labeled secondary antibody. The relative fluorescence intensity in the images was measured using ImageJ. Nuclei were stained with RedDot™1. Scale bar: 7.5 µm. (**C**) Trimerization assay. BmN cells expressing CARC mutants were harvested for non-reducing gel electrophoresis and SDS-PAGE, followed by western blotting with anti-GP64 and β-Tubulin antibodies. The relative expression of GP64 was assessed, as shown in the lower panel, by analyzing the gray intensity of Trimer1, normalized to β-Tubulin. (**D**) Cell ELISA analysis of CARC mutants. BmN cells in a 96-well plate were transfected with 0.15 µg of plasmid DNA and fixed at 72 h p.t. for cell-based ELISA analysis using a GP64 antibody. (**E**) Fusion efficiency comparison. BmN cells transiently expressing CARC4, CARC5, and CARC6 mutants were treated with varying concentrations of MβCD for 30 min. Fusion was induced by low pH for 5 min, and syncytia were recorded at 4 h post-induction. Ten random fields were analyzed, and the fusion rate was calculated as the percentage of fused nuclei relative to the total number of nuclei. Arrows indicate syncytia. Scale bar: 50 µm.

To investigate the effects of CARC mutations on fusogenicity, we expressed GP64 mutants in BmN cells and triggered cell-cell fusion with a low pH medium at 72 h post-transfection (p.t.). Mutations in CARC1 (Y49A), CARC2 (W154A), and CARC3 (W178A) abolished syncytium formation, indicating a loss of fusogenicity, which correlates with the observed reduction in viral infectivity (Fig. 2A). These mutants were transiently expressed in BmN cells. Immunofluorescence localization revealed that the CARC mutation did not affect protein localization on the plasma membrane (PM), and a comparison of fluorescence intensity showed no significant differences (Fig. 2B). Western blot analyses detected two trimer bands in the mutants and GP64 (Fig. 2C), and relative expression was further assessed by comparing the gray intensity of trimer1 and β-Tubulin (as shown by the numbers below Fig. 2C). Additionally, cell-based ELISA of CARC mutants in BmN cells showed no significant difference in expression levels compared with GP64 (Fig. 2D), suggesting that the CARC mutations did not significantly affect GP64 expression, trimerization, or PM localization. Conversely, CARC4 (Y188A), CARC5 (F235A), and CARC6 (W299A) retained partial fusogenicity but exhibited reduced viral infectivity, with fusion indices of 15.8%, 55%, and 35.5%, respectively, compared with 74.7% for wild-type GP64 (Fig. 2E). The reduced fusogenicity of CARC4, CARC5, and CARC6 mutants correlated with decreased viral infectivity, confirming that CARC1-4 are critical for BmNPV infection with wild-type GP64. Methyl-β-cyclodextrin (MβCD) is a molecule commonly used to remove cholesterol from cell membranes, and cholesterol depletion has been shown to inhibit membrane fusion (11). We found that the reduced fusogenicity of CARC4 (Y188A), CARC5 (F235A), and CARC6 (W299A) mutations can be effectively blocked by cholesterol depletion (Fig. 2E), highlighting the critical role of cholesterol in the fusion process.

### CARC2 and CARC3 are essential for virus infection with SP-cleaved GP64 (vSP^Δn^GP64)

**Fig 3:**
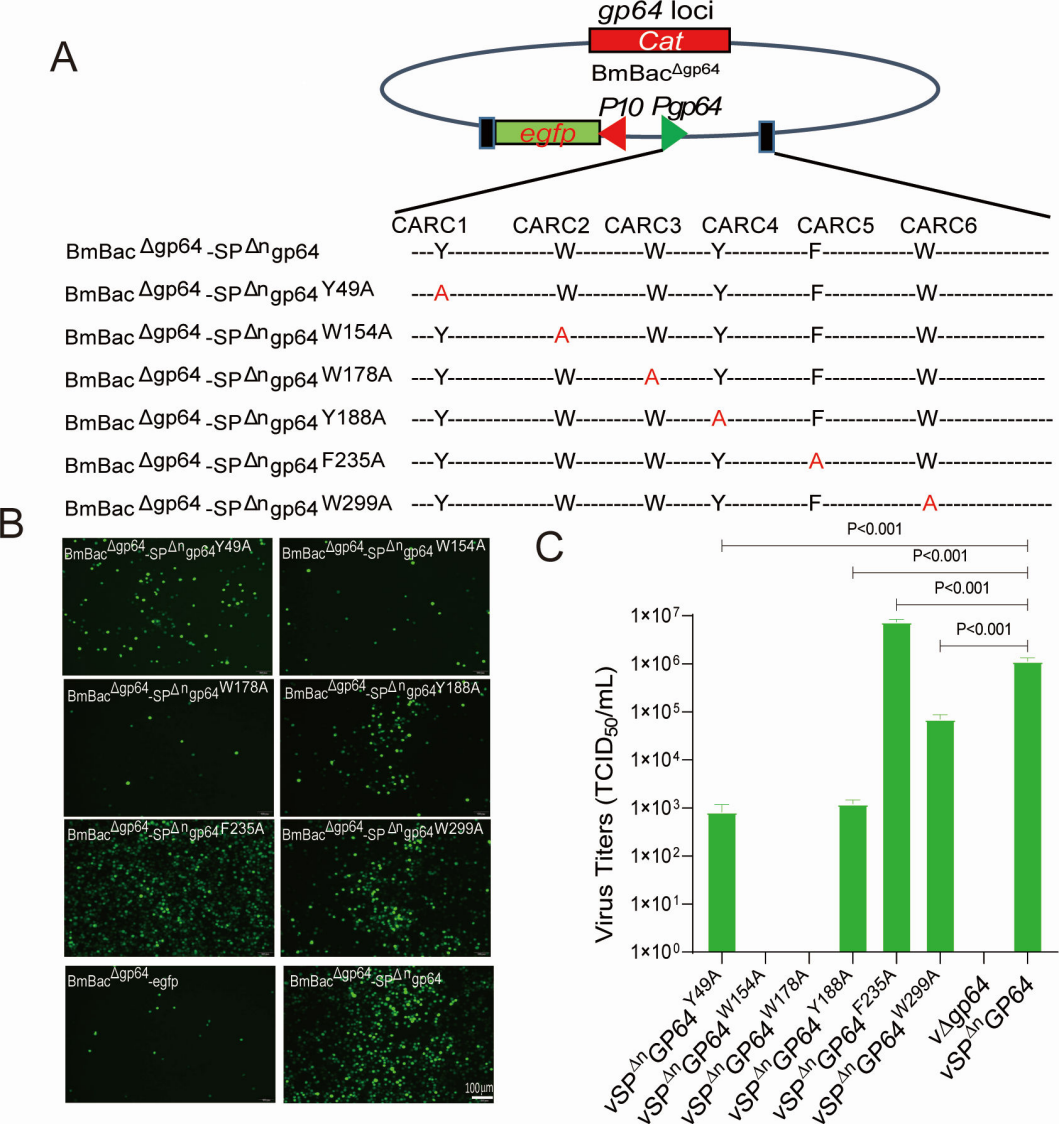
Mutation analysis of CARC in SP-cleaved GP64 (SP^Δn^GP64). (**A**) Schematic representation of CARC mutant bacmids. Aromatic amino acids in the SP-cleaved GP64 (SP^Δn^GP64) are replaced by alanine (red letters). These mutants were inserted into the *gp64*-null bacmid at the *ph* locus. (**B**) Virus replication analysis in BmN cells. Recombinant bacmids were transfected into BmN cells with H4000 transfection reagent, following the protocol. Fluorescence microscopy images were captured at 144 h p.t. Scale bar: 100 µm. (**C**) Viral titer comparison of CARC mutants. Supernatants from transfected cells were collected at 144 h p.t. for titration by EPDA.

We further explored the role of SP cleavage on CARC activity by generating six SP^Δn^GP64 mutants with CARC mutations and transposing into BmBac^Δgp64^ (Fig. 3A). Only CARC2 and CARC3 were required for infection, as their mutations failed to rescue *gp64*-null virus infectivity (Fig. 3B). BV titration revealed significantly reduced titers for vSP^Δn^GP64^W154A^ and vSP^Δn^GP64^W178A^, whereas vSP^Δn^GP64^F235A^ unexpectedly resulted in increased BV production (Fig. 3C). These results indicate that SP cleavage shifts the virus’s dependency on CARC motifs, with CARC2 and CARC3 becoming the primary drivers of fusion (Fig. 3A through C).

### CARC1 and CARC4 support cholesterol-independent virus infection

**Fig 4:**
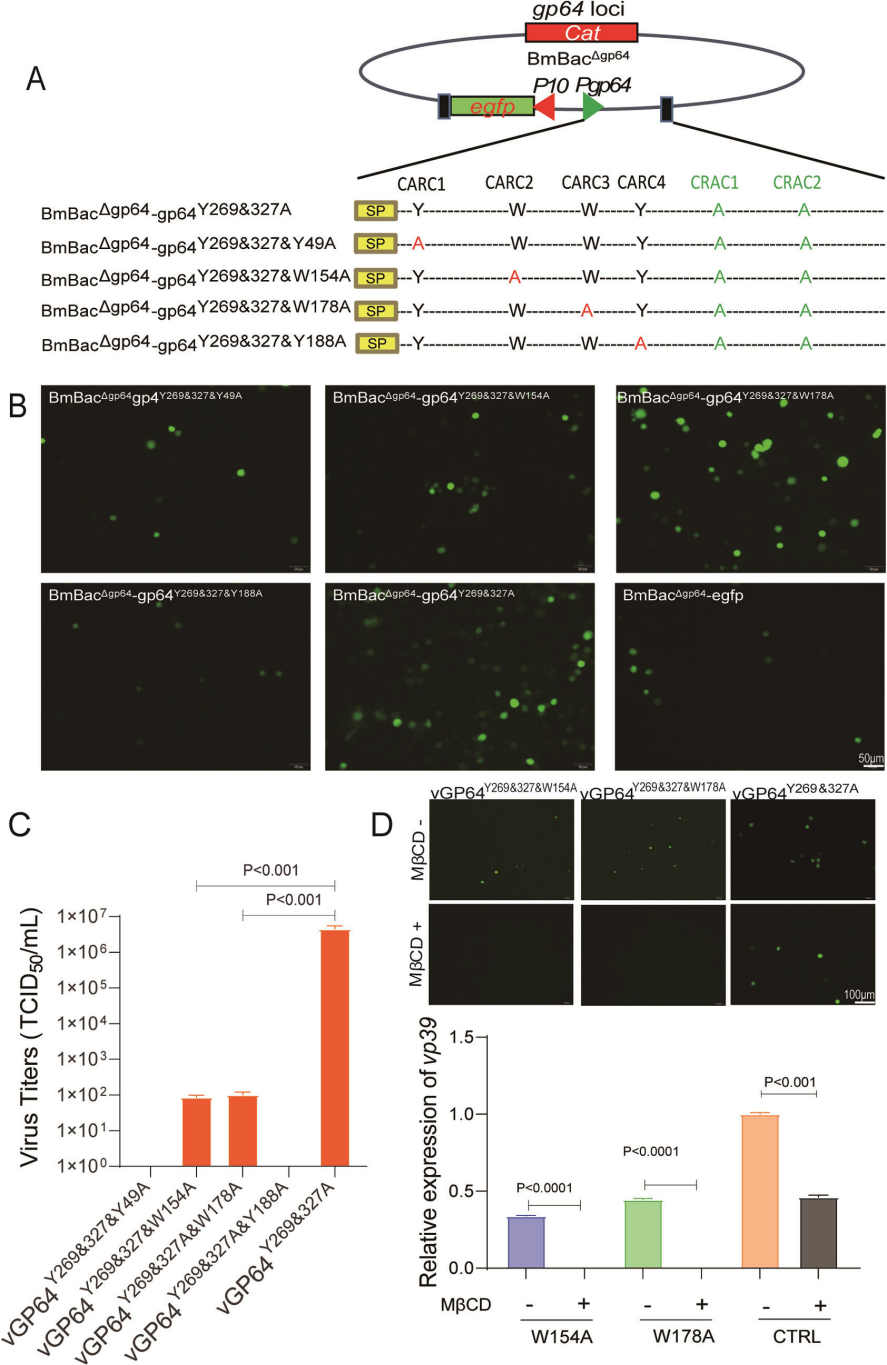
Mutation analysis of CARCs in CRAC double mutated GP64 (GP64^Y269&327A^). (**A**) Schematic representation of CARC mutant bacmids. In GP64^Y269&327A^, aromatic amino acids are replaced by alanine (red letters), with the key aromatic amino acid involved in the CRAC mutation highlighted in green. (**B**) Virus replication analysis in BmN cells. Recombinant bacmids were transfected into BmN cells using H4000 transfection reagent, following the protocol. Fluorescence microscopy images were captured at 96 h p.t. Scale bar: 50 µm. (**C**) Viral titer comparison of CARC mutants. Supernatants from transfected cells were collected at 120 h p.t. for titration by EPDA. (**D**) Effect of cholesterol depletion on viral infection. BmN cells were preincubated with 10 mM MβCD for 30 min, with PBS-treated cells serving as controls. Cells were then infected with vGP64^Y269&327A&W154A^, vGP64^Y269&327A&W178A^, or control vGP64^Y269&327A^ at an MOI of 0.1 for 2 h. Fluorescence was recorded at 36 h p.i. Scale bar: 100 µm. Subsequently, cells were harvested for RNA isolation and subjected to qPCR analysis using *vp39* primers, with *GAPDH* serving as the internal control.

Previous studies have demonstrated that BmNPV infection relies on CRAC1 and CRAC2 of GP64, any CRAC mutation resulted in the loss of viral infectivity. Notably, double mutations in CRAC1 and CRAC2 (GP64^Y269&Y327A^) restore viral infectivity, enabling the virus to bypass cholesterol dependence (9). To investigate role of CARC motif in cholesterol-independent infection, we introduced CARC mutations into GP64^Y269&327A^ (Fig. 4A). As shown in Fig. 4B, CARC1 (Y49A) and CARC4 (Y188A) mutations abolished virus infectivity, whereas CARC2 (W154A) and CARC3 (W178A) mutations resulted in significantly reduced infectivity, which was further confirmed by BV titration (Fig. 4C). To further explore the cholesterol dependence of these viruses, BmN cells treated with 10 mM MβCD and infected with the mutants or vGP64^Y269&327A^. Fluorescence amplification and qPCR analysis at 36 h post-infection (h p.i.) demonstrated that MβCD incubation completely blocked infection by the mutants vGP64^Y269&327&W154A^ and vGP64^Y269&327&W178A^, but not by vGP64^Y269&327A^ (Fig. 4D), indicating that CARC mutations restored cholesterol dependence. This suggests that CARC1 and CARC4 are vital for cholesterol-independent viral entry, highlighting the diverse functions of CARC motifs in BmNPV GP64-mediated fusion (Fig. 4A through D).

### CARC peptides affect virus infection

**Fig 5:**
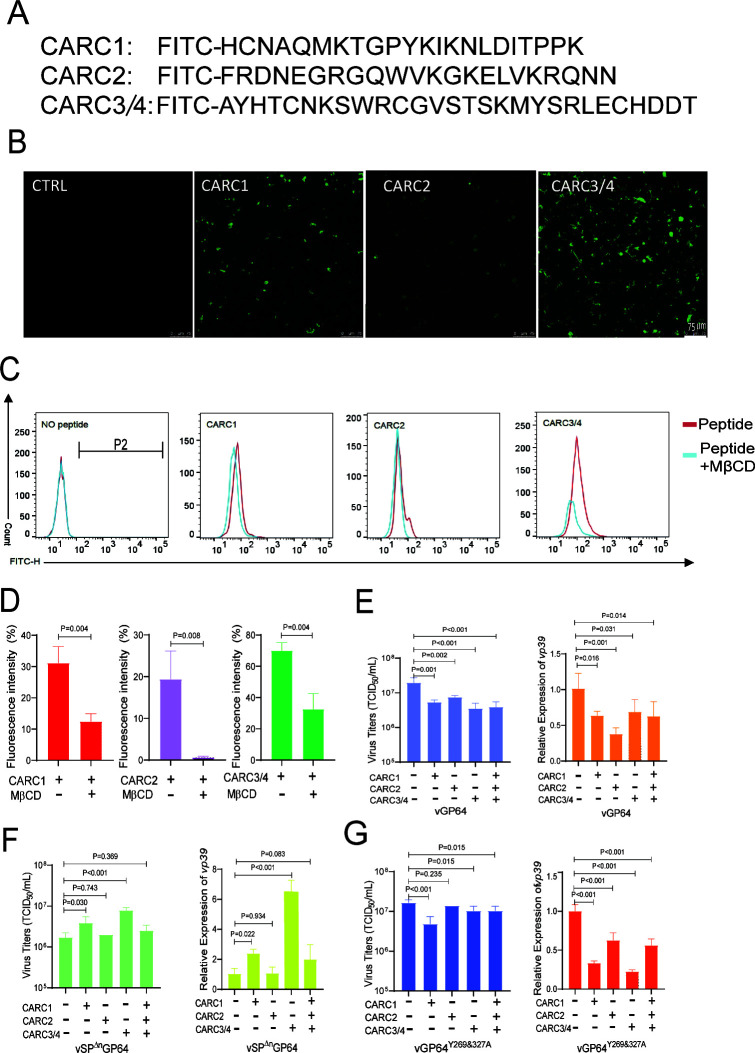
Binding assay of peptides and effect on virus infection. (**A**) CARCs sequences of GP64 labeled with FITC. (**B**) Fluorescence distribution in BmN cells. BmN cells were incubated with FITC-labeled CARC1, CARC2, and CARC3/4 peptides at a concentration of 10 µM at 27°C for 30 min. After incubation, the cells were washed twice with PBS before imaging. Scale bar: 75 µm. (**C**) Fluorescence analysis of peptides binding. BmN cells were preincubated with or without 10 mM MβCD for 30 min, followed by incubation with 10 µM peptides for 30 min. After washing with TC-100 medium, cells were analyzed by flow cytometer. (**D**) Effect of cholesterol depletion on peptides binding. Fluorescence intensity of the cells treated as in (**C**) was measured by flow cytometer, with significance indicated. Effect of peptides incubation on viral infection of vGP64 (**E**), vSP^Δn^GP64 (**F**), and GP64^Y269&327A^ (**G**). BmN cells were infected with viruses at an MOI of 5 in the presentence of FITC-labeled peptides (final concentration, 10 µM) for 2 h. Cells and supernatants were harvested at 72 h p.i. for *vp39* expression analysis by q-PCR and BV titration, with significance indicated.

To further explore the role of the CARC motif interaction with cholesterol, we synthesized fluorescein isothiocyanate (FITC)-tagged CARC peptides (Fig. 5A). Upon incubation with BmN cells, fluorescence microscopy revealed that CARC1 and CARC2 exhibited weak binding with host cells, whereas CARC3/4 displayed stronger binding (Fig. 5B). These peptides were internalized via endocytosis, suggesting that surface-bound CARC peptides can trigger this process. Flow cytometry (FCM) analysis confirmed that CARC3/4 had a stronger binding affinity than CARC1 and CARC2 (Fig. 5C and D). Incubation with 10 mM MβCD significantly reduced, but did not completely abolish, CARC binding, particularly for CARC2 (Fig. 5C and D), confirming the cholesterol-dependent nature of this interaction.

When mixed with BV vGP64, vSP^Δn^GP64, or vGP64^Y269&327A^ and used to infect BmN cells, CARC peptides significantly reduced *vp39* expression and BV titers in vGP64-infected cells, compared with the no-peptide control at 72 h p.i. (Fig. 5E). Interestingly, CARC peptides did not inhibit vSP^Δn^GP64 infection; instead, CARC1 and CARC3/4 significantly enhanced viral infection (Fig. 5F), echoing previous findings that limited cholesterol depletion enhances BmNPV infectivity (13, 31). This suggests that CARC1 and CARC3/4 may enhance vSP^Δn^GP64 entry by modulating signaling and endocytosis. Similarly, CARC peptides reduced infection by vGP64^Y269&327A^ harboring uncleaved SP (Fig. 5G), implying that SP retention alters CARC bioactivity.

### Structural insights from alphafold and SWISS-MODEL prediction

**Fig 6:**
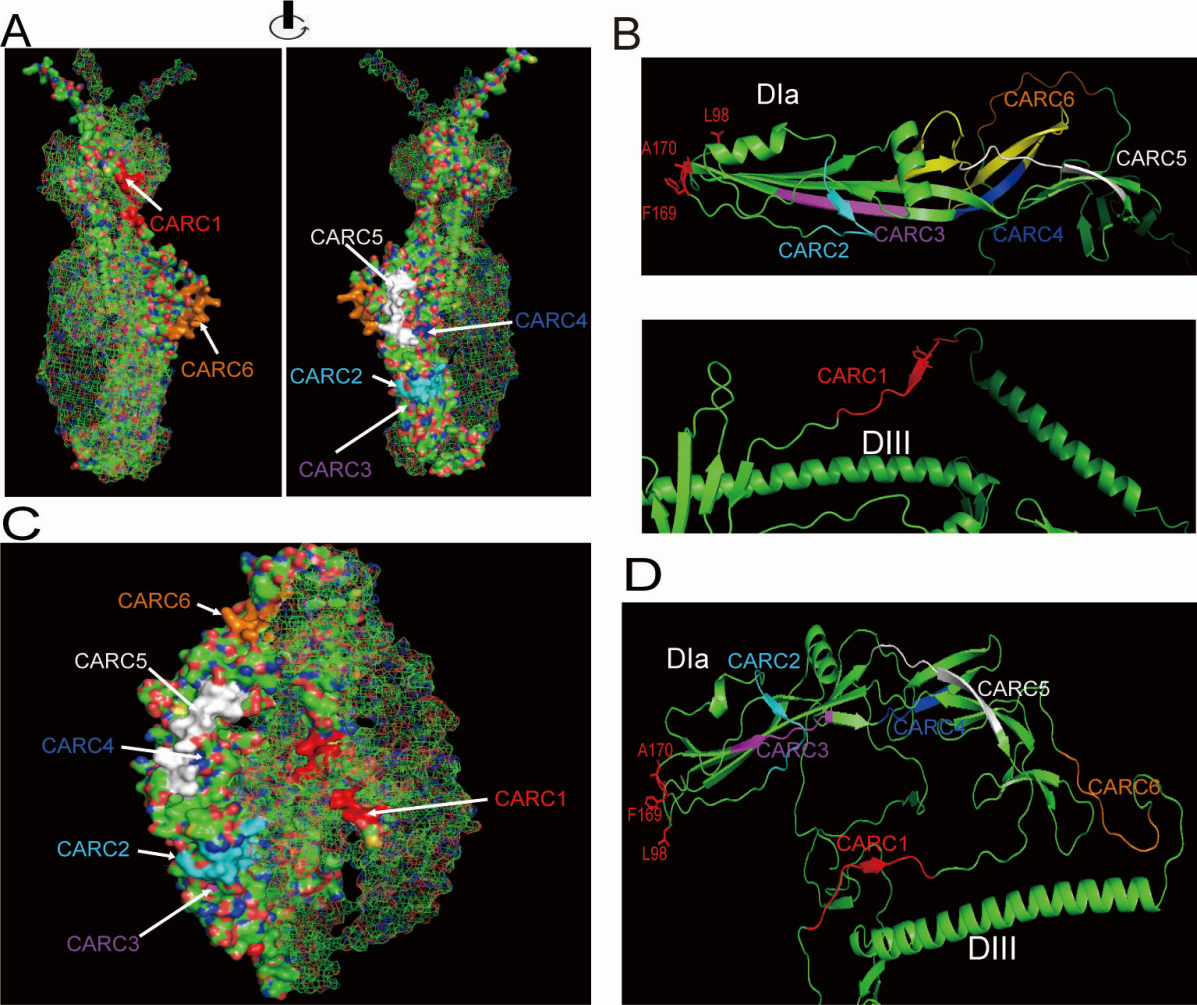
Bioinformatics prediction of CARCs localization in GP64. (**A**) Surface representation of CARCs in the GP64 trimer generated by Alphafold3. Arrows indicate the localization of the CARC motifs. (**B**) Localization of CARCs in the ribbon diagram. Key residues within the FLs are highlighted in red stick. (**C**) Surface representation of CARCs in the GP64 trimer, generated using SWISS-MODEL based on the pre-fusion structure of AcMNPV GP64. (**D**) Ribbon diagram of the pre-fusion structure showing the localization of CARC motifs, with key residues in the FLs highlighted in red sticks.

To elucidate the mechanism of CARC-mediated BmNPV infection, we predicted the tertiary structure of BmNPV GP64 with the SP using AlphaFold3 (32), based on the post-fusion structure of AcMNPV GP64. The prediction revealed that CARC1, CARC2, CARC5, and CARC6 are located on the trimer surface of GP64, whereas CARC3 and CARC4 are not exposed to the external environment (Fig. 6A). Domain analysis shows CARC2 and CARC3, situated in Domain Ia (Fig. 6B), are optimally positioned for direct interaction with cholesterol, reinforcing their role as key motifs in the fusion process. CARC1, localized in Domain III, and CARC4, in Domain Ib, appear crucial for vGP64^Y269&327A^ functionality, suggesting potential interactions with unknown viral factors. Meanwhile, CARC5 and CARC6, located in Domain II and a non-structural region, may be functionally redundant (Fig. 6B). During the review process, the pre-fusion structure (8YG6) of AcMNPV GP64 was released (33). Based on this structure, the BmNPV GP64 model was remodeled using SWISS-MODEL. As shown in Fig. 6C and D, the localization of CARCs remained consistent, with minor structure changes observed in CARC3 and CARC4, highlighting their key roles in the fusion process.

### SP retention alters virus dependence on FLs

**Fig 7:**
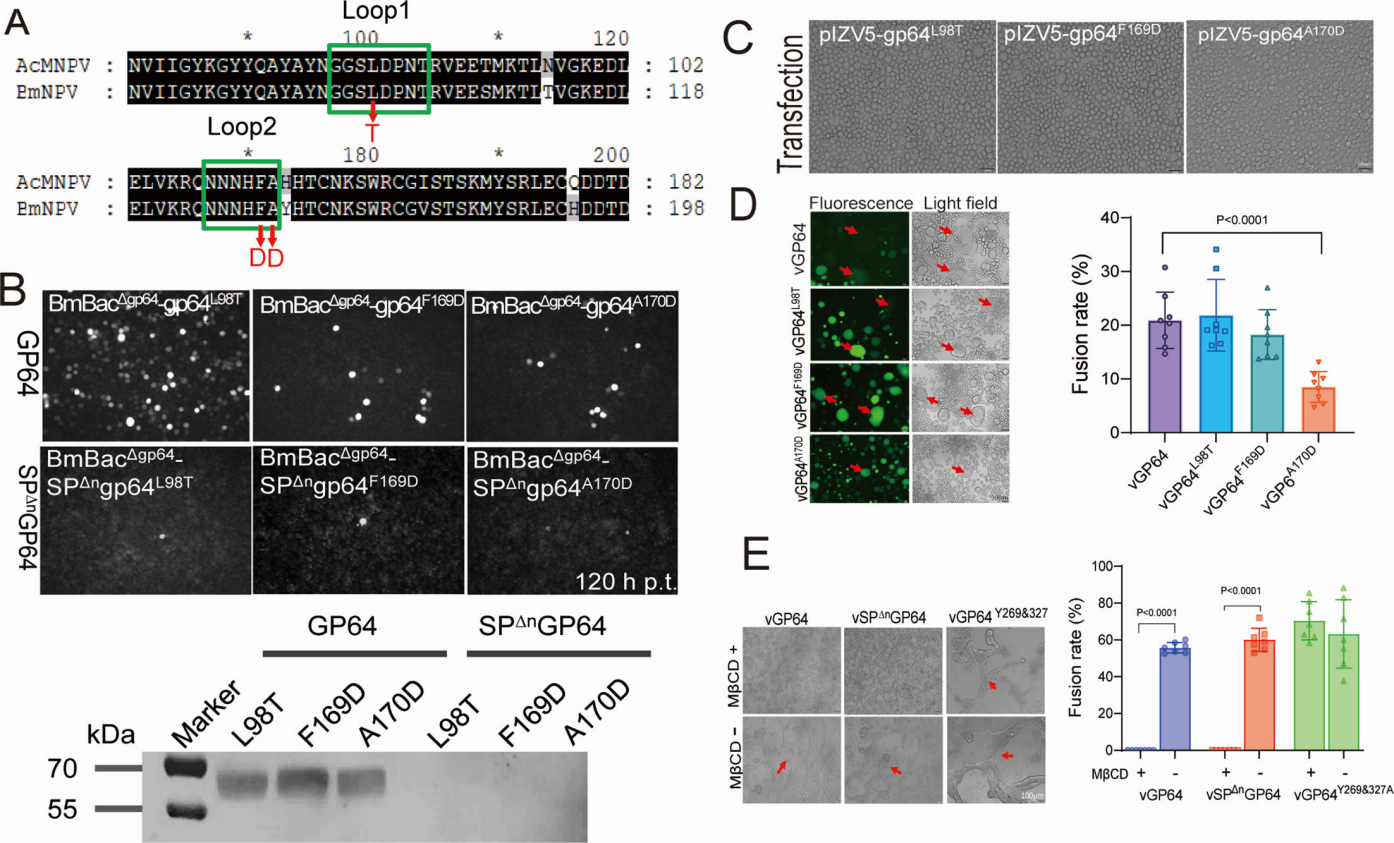
FLs analysis upon SP cleavage. (**A**) Comparison of FLs between AcMNPV and BmNPV GP64. FLs are highlighted in green boxes, with key residue mutations shown in red. (**B**) Amplification of bacmids harboring FLs mutants in BmN cells. Three key residues in *gp64* and *SP*^*Δn*^*gp64* were mutated and inserted into *gp64*-null bacmids. These bacmids were then transfected into BmN cells, and fluorescence was recorded at 120 h p.t. Cells were harvested for western blot analysis using an anti-GP64 antibody. Scale bar: 50 µm. (**C**) Fusion assay of mutants. FLs mutants of GP64 were transiently expressed in BmN cells, and fusion was induced at 72 h p.t. using a low pH medium. Scale bar: 50 µm. (**D**) Comparison of viruses with FL mutations. BmN cells were infected with vGP64, vGP64^L98T^, vGP64^F169D^, and vGP64^A170D^ at an MOI of 3, followed by incubation with a low pH medium at 72 h p.i. Syncytia are indicated by red arrows. Ten random fields were analyzed, and the fusion rate was calculated as the percentage of fused nuclei relative to the total number of nuclei. Scale bar: 50 µm. (**E**) Effect of cholesterol depletion on syncytia formation. BmN cells were infected with vGP64, vSP^Δn^GP64, and vGP64^Y269&327A^ at an MOI of 3. The cells were pretreated with 10 mM MβCD or PBS for 30 min at 36 h p.i., followed by incubation with a low pH medium to induce syncytium formation. Syncytia are indicated by red arrows. Ten random fields were analyzed, and the fusion rate was calculated as the percentage of fused nuclei relative to the total number of nuclei. Scale bar: 100 µm.

GP64-mediated membrane fusion relies heavily on two FLs within Domain Ia, which are conserved between BmNPV and AcMNPV GP64 (Fig. 7A). Notably, CARC2 and CARC3 are strategically located on either side of the FL; the retention of the SP might induce conformational changes in these motifs, potentially affecting their interaction with cholesterol and, consequently, their role in mediating fusion. To investigate the role of FLs in BmNPV GP64, we generated three key residue mutants—GP64^L98T^, GP64^F169D^, and GP64^A170D^—in both GP64 and SP^Δn^GP64. As expected, mutation on vSP^Δn^GP64 abolished infectivity (Fig. 7B), underscoring the critical role of these residues in membrane fusion. Interestingly, the same mutations on GP64 did not eliminate the infectivity of the recombinant viruses. Although these mutations inhibited cell-cell fusion in transient expression assays (Fig. 7C), syncytia formation was still observed in cells infected by these viruses upon low pH treatment (Fig. 7D), and vGP64^A170D^ exhibited weaker fusion ability than vGP64, suggesting that these residues, although not essential for viral infectivity, play a significant role in cell-cell fusion, potentially involving unknown viral factors.

Notably, transient expression of SP^Δn^GP64 failed to localize to the PM, thereby preventing cell-cell fusion under these conditions. However, during infection, SP^Δn^GP64 was successfully transported to PM (29). To further investigate the role of cholesterol in the fusion, we compared cholesterol dependence of vGP64, vSP^Δn^GP64, and vGP64^Y269&327A^. As expected, all three viruses formed syncytia upon low pH exposure (Fig. 7E). However, MβCD incubation inhibited fusion in both vSP^Δn^GP64 and vGP64 but did not significantly affect vGP64^Y269&327A^, indicating that SP^Δn^GP64 and wt-GP64 mediate fusion in a cholesterol-dependent manner, whereas GP64^Y269&327A^ mediates fusion independently of cholesterol.

## DISCUSSION

Our findings reveal a sophisticated fusion mechanism in BmNPV GP64, where CARC motifs play dual roles depending on SP retention. Specifically, CARC2 and CARC3 are critical for cholesterol-dependent fusion, whereas CARC1 and CARC4 may facilitate an alternative, cholesterol-independent pathway. This adaptability likely enhances the virus’s ability to infect host cells, even under varying membrane compositions, highlighting the versatility of the BmNPV fusion apparatus. The discovery that double CRAC mutations in BmNPV GP64 can restore infectivity in a cholesterol-independent manner via CARC1 and CARC4 underscores the potential of targeting these motifs in antiviral strategies. This dual-pathway model not only advances our understanding of BmNPV biology but also suggests that similar mechanisms could be at play in other enveloped viruses, expanding the potential implications of our findings.

**Fig 8:**
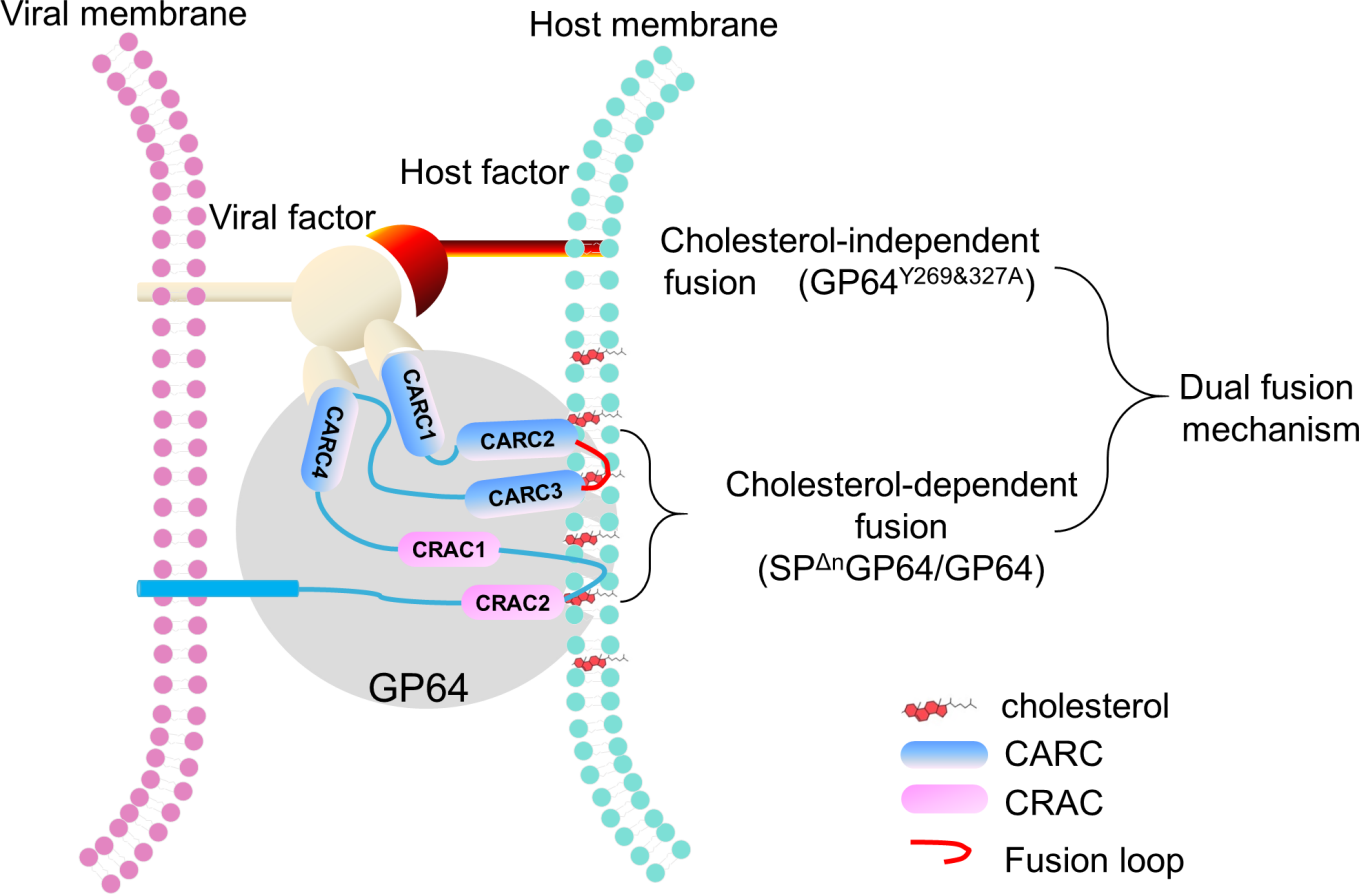
Proposed model of GP64 mediate membrane fusion.

Based on our studies (Table 1) (9, 10, 28, 29, 34), we propose a model in which the retention of the SP in GP64 facilitates a flexible fusion strategy that engages all four CARC motifs—CARC1, CARC2, CARC3, and CARC4—and two CRAC motifs—CRAC1 and CRAC2—in membrane fusion (Fig. 8). Upon SP cleavage, the fusion process becomes primarily reliant on CARC2, CARC3, and CRAC2, which are located in Domain Ia and helix B. These motifs are crucial for maintaining GP64’s structural integrity during membrane fusion and are involved in cholesterol-dependent on fusion, the primary mechanism employed by GP64. Notably, when double mutations occur in CRAC1 and CRAC2, impairing CARC2 and CARC3, the virus compensates by utilizing CARC1 and CARC4 in a cholesterol-independent manner, demonstrating the resilience and adaptability of the BmNPV fusion mechanism.

**TABLE 1:** Summary of mutations on fusogencity and infectivity^*a*^

Mutation	GP64		SP^Δn^GP64	GP64^Y269&327A^
	Infectivity	Fusogencity		
Y49A	-	-	+	-
W154A	-	-	-	+
W178A	-	-	-	+
Y188A	-	+	+	-
F235A	+	+	+	/
W299A	+	+	+	/

^*a*^ Fusogenencity represents the fusion ability of transient expression of mutants in BmN cells.

The ability of GP64 to toggle between cholesterol-dependent and -independent fusion pathways illustrates the evolutionary sophistication of BmNPV. This versatility is exemplified by the restored infectivity of the CRAC double-mutant GP64 (vGP64^Y269&327A^) (9), highlighting the critical balance between CRAC and CARC motifs. These findings suggest that the virus can maintain fusion integrity by modulating interactions with cholesterol, a strategy that might be conserved across other viral fusion proteins (14).

Our model builds on existing knowledge of viral fusion mechanisms (35), offering a nuanced understanding of how BmNPV adapts to its host’s membrane environment. This adaptability is similar to the functional compensation observed in both RNA (36–39) and DNA viruses (40, 41), which enables them to thrive in varying host conditions. Although other viruses like Influenza and HIV heavily rely on cholesterol for membrane fusion (21, 42, 43), BmNPV demonstrates a unique ability to bypass this dependency, positioning it as a model for studying alternative fusion pathways that could reveal new targets for antiviral intervention.

The dual mechanisms identified in this study open new avenues for antiviral strategy development. Targeting the cholesterol interaction sites on CARC2 and CARC3 could disrupt the primary fusion pathway, whereas simultaneously inhibiting the cholesterol-independent mechanisms may enhance antiviral efficacy. Future research should further investigate the structural dynamics of GP64, particularly in response to SP cleavage, and explore whether similar mechanisms are employed by other enveloped viruses.

Although our study provides substantial insights into the fusion mechanisms of BmNPV, further research is needed to fully elucidate the roles of other lipid components in the fusion process. Expanding this research to other viral fusion proteins could determine whether the dual-pathway mechanism we propose is a common feature among viruses. The broader impact of our findings lies in their potential to reshape the current understanding of viral entry and to inform the design of novel antiviral strategies targeting these critical fusion motifs.

In conclusion, our proposed fusion model emphasizes the complexity and adaptability of the BmNPV fusion mechanism, driven by the intricate interplay of CARC motifs. This model not only enhances our understanding of viral fusion but also provides a robust framework for the development of innovative antiviral therapies.

## MATERIALS AND METHODS

### Cell and bacmids

BmN cells were cultured in a TC-100 insect medium (AppliChem, Darmstadt, Germany) supplemented with 10% FBS (Gibco BRL, Gaithersburg, MD, USA) using standard techniques. *Gp64*-null bacmid (BmBac^Δgp64^), BmBac^Δgp64^-gp64, BmBac^Δgp64^-SP^Δn^gp64, and BmBac^Δgp64^-gp64^Y269&327A^ were previously constructed (44, 45).

### Generation of CARC mutants

CARC mutants were generated using specific PCR primers (Table 2) to replace key aromatic residues (tyrosine, tryptophan, or phenylalanine) with alanine. These mutant genes (*gp64*^Y49A^, *gp64*^W154A^, *gp64*^W178A^, *gp64*^Y188A^, *gp64*^F235A^, *gp64*^W299A^, *SP^Δn^gp64*^Y49A^, *SP^Δn^gp64*^W154A^, *SP^Δn^gp64*^W178A^, *SP^Δn^gp64*^Y188A^, *SP^Δn^gp64*^F235A^, *SP^Δn^gp64*^W299A^; *gp64*^Y269&327&Y49A^, *gp64*^Y269&327&W154A^, *gp64*^Y269&327&W178A^, and *gp64*^Y269&327&Y188A^) were cloned into a pFBD-egfp transfer vector via *Eco*R I and *Xba* I digestion and transposed into BmBac^Δgp64^ to produce recombinant bacmids (9).

**TABLE 2:** Primers used in this study

Name	Sequence (5’ - 3’)
Pro/gp64-F	CGCGAATTCGACAGATATTTAAATAAACCAAAC
gp64-R	GCGTCTAGATTAATATTGTCTACTATTACGGTT
Y49A-F	AATGAAAACGGGTCCGGCCAAAATTAAAAACTTG
Y49A-R	CAAGTTTTTAATTTTGGCCGGACCCGTTTTCATT
W154A-F	CGAAGGCCGCGGCCAGGCGGTCAAAGGCAAAGAG
W154A-R	CTCTTTGCCTTTGACCGCCTGGCCGCGGCCTTCG
W178A-F	CACGTGCAACAAATCGGCGCGATGCGGCGTTTCT
W178A-R	AGAAACGCCGCATCGCGCCGATTTGTTGCACGTG
Y188A-F	TTCTACTTCGAAAATGGCCAGCAGGCTCGAGTGC
Y188A-R	GCACTCGAGCCTGCTGGCCATTTTCGAAGTAGAA
F235A-F	CAAACAAAAGTCTACGGCCACCACGCGCCAAGTA
F235A-R	TACTTGGCGCGTGGTGGCCGTAGACTTTTGTTTG
W299A-F	GAAACGACCGCCCACTGCGCGTCACAACGTTAGA
W299A-R	TCTAACGTTGTGACGCGCAGTGGGCGGTCGTTTC
M13-F	GTTGTAAAACGACGGCCAG
eGFP-F	GGCCCCGGGATGGTGAGCAAGGGCGAGG
L98T-F	TACAACGGAGGCTCGACGGATCCCAACACACGC
L98T-R	GCGTGTGTTGGGATCCGTCGAGCCTCCGTTGTA
F169D-F	CAGAATAACAATCACGATGCGTACCACACGTGC
F169D-R	GCACGTGTGGTACGCATCGTGATTGTTATTCTG
A170D-F	AATAACAATCACTTTGACTACCACACGTGCAAC
A170D-R	GTTGCACGTGTGGTAGTCAAAGTGATTGTTATT

### Transfection of recombinant bacmids

BmN cells (3 × 10^5^ cells per 35 mm well) were transfected with 2.0 µg of the respective bacmid DNA using 10 µL Entranster-H4000 (Engreen Biosystem, Beijing, China) and incubated for 4 h. After removing the transfection mixtures, cells were cultured in 2 mL fresh medium containing penicillin/streptomycin (50 units/mL and 50 µg/mL). Fluorescence was recorded by microscopy at 96 h p.t.. BVs and cell samples were harvested at 120 h p.t. for titration by endpoint dilution assay (EPDA) and western blot analysis.

### Western blot analysis

Transfected cells were lysed in 50 µL of PBS (pH 7.4), and 10 µL samples were separated on 10% reducing or nonreducing polyacrylamide gels, then transferred onto a 0.45 µm PVDF membrane (Millipore, USA). Primary antibodies against GP64 (AcV5, Santa Cruz, CA, USA), GFP (SABC, Shanghai, China), and β-Tubulin (SABC, Shanghai, China) were used, followed by an alkaline phosphatase-conjugated goat anti-rabbit secondary antibody (SABC, Shanghai, China). Signals were detected using NBT and BCIP (SABC, Shanghai, China).

### Construction of transient expression vector and syncytium formation assay

*Gp64* and its mutants (*gp64*^Y49A^, *gp64*^W154A^, *gp64*^W178A^, *gp64*^Y188A^, *gp64*^F235A^, and *gp64*^W299A^) were inserted into pIZ/V5-His transient expression vector using *Eco*R I and *Xba* I digestion. These plasmids were transfected into BmN cells using Entranster-H4000. Cell-cell fusion was induced with a low pH TC-100 medium (pH 4.5) at 72 h p.t. for 5 min, followed by 4 h of culture in a normal TC-100 medium. In parallel, transfected cells were preincubated with 0 mM, 4 mM, or 8 mM MβCD (Sigma Aldrich, USA) for 30 min and then induced with a low pH medium. Nuclei were stained with Hoechst 33258 (Invitrogen, Carlsbad, USA) and imaged for fusion activity measurement (29).

### Localization assay

Transient expression vectors (pIZ/V5-gp64^Y49A^, pIZ/V5-gp64^W154A^, pIZ/V5-gp64^W178A^, pIZ/V5-gp64^Y188A^, pIZ/V5-gp64^F235A^, and pIZ/V5-gp64^W299A^) were transfected into BmN cells cultured in a confocal dish (Nest Biotech, Wuxi, China). At 72 h p.t., cells were fixed with 4% paraformaldehyde for 15 min, washed with PBS (pH 7.4), and labeled with anti-GP64 antibody for 2 h. After washing, a FITC-labeled secondary antibody was applied for 1 h. Nuclei were stained with RedDot™1 (Biotium, CA, USA). Images were captured using a confocal laser scanning microscope (Leica SP8, Wetzlar, Germany), and the relative fluorescence intensity of the cells was analyzed with ImageJ software.

### Cell-based ELISA

Cell-based ELISA was performed as previously described (29) with minor modifications. BmN cells (2.5 × 10^4^) in 96-well plates were transfected with 0.15 µg transient expression vectors and fixed with 4% paraformaldehyde for 15 min, without permeabilization. The cells were blocked and incubated with GP64 antibody for 4 h, followed by washing and incubation with horseradish peroxidase (HRP)-labeled secondary antibody (Beyotime, Shanghai, China) for 1 h. After three washes with TBST, 100 µL of tetramethylbenzidine chromogen solution was added and incubated for 10 min in the dark. Color development was stopped by adding 100 µL of 2 M H_2_SO_4_ per well, and absorbance was measured using a Perkin-Elmer multimode plate reader (Waltham, MA, USA). The experiment was performed in triplicate, and data were analyzed using one-way ANOVA in GraphPad.

### MβCD incubation on viral infections and cell-cell fusion

BmN cells were seeded in 24-well plates overnight and then incubated with 10 mM MβCD for 30 min, PBS-treated cells served as controls. Cells were then infected with vGP64^Y269&327A&W154A^, vGP64^Y269&327A&W178A^, or control vGP64^Y269&327A^ at a multiplicity of infection (MOI) of 0.1 for 2 h. After removing the virus-containing medium, cells were washed twice with TC-100 medium and cultured routinely. Fluorescence was recorded at 36 h p.i. by fluorescence microscopy. Subsequently, cells were harvested for qPCR analysis. Parallel BmN cells were infected with vGP64, vSP^Δn^GP64, or vGP64^Y269&327A^ at an MOI of 3. The cells were incubated with 10 mM MβCD or PBS for 30 min at 36 h p.i. and incubated with a low pH medium to induce syncytium formation. Ten random fields were analyzed, and the fusion rate was calculated as the percentage of fused nuclei relative to the total number of nuclei.

### Peptides binding assay

Three FITC-labeled peptides (CARC1, CARA2, and CARC3/4) were synthesized (SABC, Shanghai, China). BmN cells were seeded in 24-well plates, preincubated with/without 10 mM MβCD for 30 min, and then incubated with peptides (final concentration 10 µM) for 30 min. After washing with TC-100 medium, cells were observed under a fluorescence microscope and analyzed by ACSVerse Flow Cytometer (BD, New Jersey, USA).

### Effect of peptides on virus infection

BmN cells seeded in 24-well plates were infected with vGP64, vSP^Δn^GP64, and vGP64^Y269&327^ at an MOI of 5 in the presentence of FITC-peptides (final concentration, 10 µM) for 2 h. Cells and supernatants were harvested at 48 h p.i. for *vp39* expression analysis by q-PCR and BV titration.

### FLs mutation analysis

Key residue mutation in the FLs (*gp64*^L98T^, *gp64*^F169D^, *gp64*^A170D^, *SP^Δn^gp64*^L98T^, *SP^Δn^gp64*^F169D^, and *SP^Δn^gp64*^A170D^) were generated by PCR using primers listed in Table 2. These mutants were transposed into *gp64*-null bacmid to generate recombinant bacmids, which were then transfected into BmN cells. For transient expression, *gp64*^L98T^, *gp64*^F169D^, and *gp64*^A170D^ mutants were inserted into pIZ/V5-His vector. Cell-cell fusion was induced with a low-pH medium at 72 h p.t. For the fusion assay of the infected cells, BmN cells were infected with vGP64, vGP64^L98T^, vGP64^F169D^, and vGP64^A170D^ at an MOI of 3, followed by incubation with low pH medium at 72 h p.i. Ten random fields were analyzed, and the fusion rate was calculated as the percentage of fused nuclei relative to the total number of nuclei.

## Data Availability

All data generated or analyzed during this study are included in the published article and are available from the corresponding author upon reasonable request.
